# Improved methods for fluorescent labeling and detection of single extracellular vesicles using nanoparticle tracking analysis

**DOI:** 10.1038/s41598-019-48181-6

**Published:** 2019-08-23

**Authors:** Kristen E. Thane, Airiel M. Davis, Andrew M. Hoffman

**Affiliations:** Department of Clinical Sciences, Cummings School of Veterinary Medicine, Tufts University, Massachusetts, USA

**Keywords:** Diagnostic markers, Fluorescence imaging, Biomarkers

## Abstract

Growing interest in extracellular vesicles (EV) has necessitated development of protocols to improve EV characterization as a precursor for myriad downstream investigations. Identifying expression of EV surface epitopes can aid in determining EV enrichment and allow for comparisons of sample phenotypes. This study was designed to test a rigorous method of indirect fluorescent immunolabeling of single EV with subsequent evaluation using nanoparticle tracking analysis (NTA) to simultaneously determine EV concentration, particle size distribution, and surface immunophenotype. In this study, EV were isolated from canine and human cell cultures for immunolabeling and characterized using NTA, transmission electron microscopy, and Western blotting. Indirect fluorescent immunolabeling utilizing quantum dots (Qd) resulted in reproducible detection of individual fluorescently labeled EV using NTA. Methods were proposed to evaluate the success of immunolabeling based on paired particle detection in NTA light scatter and fluorescent modes. Bead-assisted depletion and size-exclusion chromatography improved specificity of Qd labeling. The described method for indirect immunolabeling of EV and single vesicle detection using NTA offers an improved method for estimating the fraction of EV that express a specific epitope, while approximating population size distribution and concentration.

## Introduction

Studies of extracellular vesicle (EV) biogenesis, disease relevance, and diagnostic and therapeutic potential have rapidly expanded over the last decade, prompting the advancement of rigorous methods to characterize the biophysical, phenotypic, and functional attributes of EV^1^. Characterization of EV, individually and in aggregate, at a minimum includes estimations of particle size distribution and detection of EV-associated proteins such as CD9, CD63, CD81, Alix, and TSG101^2–5^. More extensive phenotypic analyses utilize immunoblotting, ELISA, or single–vesicle or bulk immunolabeling of surface epitopes that discriminate unique repertoires of EV^6–9^. Recent focus on the role of EV cargo^10^, particularly EV-associated protein^11–13^, RNA^14–16^, lipids^17,18^, and DNA^19,20^, has emphasized the importance of accurate characterization of EV enrichment of samples, as non-EV contaminants may lead to misinterpretation of experimental results^21,22^. This has spurred development of techniques that accurately assess the degree of EV enrichment of samples^23–25^. However, several obstacles exist to quantifying enrichment, such as “universal labeling” of all EV within a sample. For example, recent studies^26,27^ demonstrate that fluorescent detection of EV labeled with lipophilic dyes may be confounded by nonspecific labeling of particles (lipoprotein or protein sample contaminants) or by the presence of non-EV particles within the labeling mixture. Thus far, a method combining protein detection on single vesicles with measurements of bulk particle size distribution and concentration has been elusive.

Nanoparticle tracking analysis (NTA) is a widely employed technique for characterization of particle size distribution of EV. NTA tracks the Brownian motion of individual particles, enabling size determinations of single EV and populations of EV using the Stokes-Einstein equation^28,29^. NTA utilizes laser light for particle interrogation, ostensibly enabling specific fluorochrome excitation and detection of fluorescently labeled EV (“NTA fluorescence” or NTA-FL herein). There are few reports demonstrating rigorous assessment of NTA-FL, and those which exist in the literature have relied upon immunolabeling non-canonical EV protein targets^30–32^ or non-specific labeling^33^. These reports demonstrate the potential of this application and due to the instability of many fluorophores has led to the use of quantum dots for application with NTA-FL. Quantum dots (Qd) are semiconductor nanocrystals that are 10–30 nm in diameter, bright, photostable, and feature broad ranges of excitation and emission wavelengths with large Stokes shifts^34,35^, features ideally suited for NTA applications.

However, NTA-FL using Qd has been plagued with several additional obstacles, including diminished reactivity of antibodies when directly conjugated with Qd (markedly limiting the range of antibodies that can be tested) and the absence of effective protocols to separate Qd from Qd-labeled EV during indirect immunolabeling protocols. The purpose of this study was to test a method for indirect fluorescent immunolabeling of single EV for NTA-FL that overcomes these shortfalls, facilitating single–vesicle immunolabeling and detection concurrent with particle size distribution and concentration. We demonstrate that a combination of indirect immunolabeling utilizing Qd coupled with size-exclusion chromatography offers a robust method for exploiting NTA-FL for this purpose.

## Methods

### EV production

Canine mesenchymal stromal cells (MSCs) of placental origin and human embryonic kidney cells ATCC CRL-1573 (HEK-293) were used to generate EV for immunolabeling experiments. Cells were grown to sub-confluence (70–80%) in alpha-MEM (aMEM, Lonza) with 15% fetal bovine serum (FBS, Hyclone) for MSCs or 10% FBS for HEK-293, plus 2mM L-glutamine (Lonza), 100 units/mL penicillin (Hyclone), and 100 μg/mL streptomycin (Hyclone). Cultures were washed of growth medium using commercial phosphate-buffered saline (PBS, Lonza) and then switched to serum-free defined chemical medium (DCM) containing DMEM (Gibco) supplemented with ITS (10 μg/mL insulin, 10 μg/mL transferrin, 10 ng/mL selenium; Lonza), 5 ng/mL FGF2 (Invitrogen), 5 ng/mL PDGF (Invitrogen), 25 mM HEPES (Gibco), 2 mM L-glutamine, 100 units/mL penicillin, and 100 μg/mL streptomycin. The conditioned DCM was removed from the cell culture after 48 hours for EV isolation.

### EV isolation

The EV isolation method was based on prior published methods^3^. Briefly, EV were isolated by stepwise ultracentrifugation performed using an Optima L-90K equipped with fixed-angle 45 Ti (k factor 133) and 70.1 Ti (k factor 36) rotors (Beckman Coulter). Cell-conditioned DCM was centrifuged at 300 *g* for 10 minutes, 2,000 *g* for 10 minutes, and 10,000 *g* for 30 minutes to remove cell debris, apoptotic bodies, protein aggregates, and larger vesicles (microvesicles), with the supernatant retained at each step. The final supernatant was ultracentrifuged at 100,000 *g* for 2 hours using a 70.1 Ti rotor and the resultant pellet was resuspended in PBS for NTA and subsequent immunolabeling. All EV samples were stored at 4 °C until immunolabeling could be performed (0–4 days following isolation).

### NTA protocol

All particle tracking analyses were performed using a NS300 unit (Malvern) equipped with a 488 nm laser and a 500 nm long-pass filter for fluorescence detection. All samples were diluted to provide a concentration of 1 × 10^8^–1 × 10^9^ particles/mL counted using NTA. All counts were performed in replicates of 5 for each sample, collecting 30–60-second videos with a minimum of 200 valid tracks recorded per video (minimum of 1000 valid tracks recorded per sample). Nanosight 3.0 software was used for all analyses, using standard settings. The camera level for each sample was manually adjusted to achieve optimal visualization of particles^36^. For all experiments, the camera level setting ranged from 12–14 for samples analyzed in light scatter mode (LSM) and from 15–16 for samples analyzed in fluorescence mode (FM). Detection threshold (DT) was set for maximum sensitivity with a minimum of background noise, with the level ranging from 5–7 for samples analyzed in LSM and from 3–4 for samples analyzed in FM. The sample infusion pump was set to a constant flow rate of 5 μL/minute. To minimize variability, all camera and detection threshold settings were kept the same for each mode when performing multiple experiments on a single sample source. To minimize photobleaching for FM, all immunolabeled samples were evaluated first in FM, followed immediately by evaluation in LSM. Validity of reported particle size was periodically assessed for the NS300 unit using 100 nm and 200 nm polystyrene beads (Malvern, Spherotech).

### Antibody labeling of EV

The following antibodies were used for immunolabeling: anti-CD9 [MM2/57] (ab19761), anti-CD9-biotin [MM2/57] (ab34161), anti-CD9-Qd655 conjugate [MM2/57] (ab19761; SiteClick Qd labeling kit, Thermo Fisher), anti-CD81 [Clone JS-81] (BD 555675), anti-CD81-APC [Clone JS-81] (BD 551112), anti-CD81-biotin [Clone JS-81] (BD 555675; EZ-Link Micro Sulfo-NHL-biotinylation kit, Thermo Fisher), IgG_2b_-biotin isotype (BD 559531). Qd655-streptavidin (Q10121MP) or Donkey anti-Mouse IgG-Qd655 (Q22088) was used for secondary labeling.

### Preliminary EV labeling

Bulk labeling of EV adsorbed to polystyrene beads was performed and analyzed using flow cytometry to evaluate antibody function (Supplementary information part 1). Varying concentrations of EV were labeled in bulk to determine an optimized antibody to EV ratio for single-vesicle labeling (Supplementary Information part 2). Additionally, liposome standards and EV were labeled using an ExoGlow labeling kit following the manufacturer’s instructions and evaluated using NTA-FL (Supplementary Information part 3).

### Single EV immunolabeling and NTA evaluation

Particle concentrations were established for each unlabeled EV sample prior to immunolabeling. A volume containing 1 × 10^10^ particles (as counted by NTA) was incubated with 1 μg or “one test” of Ab with PBS to yield a total volume of 100 μL for incubation at 4 °C overnight. Ultracentrifugation at 100,000 *g* for 2 hours was repeated on the labeled sample as a wash step to separate labeled EV from unbound Ab. The washed, primary Ab-labeled EV pellet was resuspended to a final volume of 50–100 μL in PBS. Optimization of Qd655-SAV volume was evaluated using varying amounts of Qd with CD9-biotin labeled EV (Supplementary Information part 4). A volume of 0.5–1 μL of Qd655-SAV (equivalent to 3–6 × 10^11^ Qd) was added to the primary Ab-labeled samples and incubated in darkness for 30 minutes at 4 °C. The labeled samples were immediately analyzed using NTA.

### EV immunolabeling in the presence of non-EV particles

Unconditioned cell culture growth medium containing αMEM and 15% FBS (Hyclone) was ultracentrifuged at 100,000 *g* for 18 hours to markedly deplete EV per published recommendations^37^. An aliquot of this EV-depleted, FBS-containing medium was subsequently ultracentrifuged as described for EV isolation; the resultant pellet was resuspended in sterile PBS to be used as a source of non-EV “contaminant” particles. The FBS-derived particle concentration was established using NTA. Samples containing isolated EV alone and a 1:1 ratio of EV mixed with FBS-derived particles were immunolabeled as described above and subsequently analyzed on NTA.

### Western blotting

Protein extraction was performed using mPER (Pierce) with resultant protein concentration evaluated by BCA (Pierce) following the manufacturer’s instructions. EV aliquots containing approximately 1 × 10^11^ particles were incubated in an equal volume of mPER. For each EV sample, 20 million “parent” cells (that had generated the EV) were resuspended in 1 mL of mPER. Protein samples were frozen at −20 °C until used. 4 μg of EV-derived or matched cell-derived protein was separated using Bolt 4–12% Bis-Tris gels and MES SDS running buffer (ThermoFisher). Protein was transferred onto a nitrocellulose membrane using an iBlot 2 dry blotting system (ThermoFisher) set to 15 V for 15 minutes. Ab labeling was performed using the iBind Flex system (ThermoFisher) using primary mouse anti-CD9 [MM2/57] (BioRad MCA469GA) or mouse anti-TSG101 (BD 612696) and anti-mouse IgG (Vector) secondary Ab. Positive bands were detected using the Vectastain ABC HRP kit and DAB peroxidase substrate kit (Vector) per the manufacturer’s instructions.

### Advanced microscopy

Advanced microscopy was performed to directly visualize immunolabeled EV and look for any evidence of EV aggregation associated with the immunolabeling technique. Atomic force microscopy was performed on an EV sample immunolabeled as described above. FastScan AFM (Bruker) was used in peak force mode to evaluate sample regions of 5 μm and 2.7 μm. Transmission electron microscopy (FEI Tecnai Spirit 12) was performed on samples labeled with anti-CD9-biotin and streptavidin-gold (Cytodiagnostics) using uranium acetate negative staining.

### Biotinylated bead-assisted depletion of free Qd

EV samples were prepared and immunolabeled as described for NTA evaluation. Biotinylated polystyrene microspheres (740 nm, 1% w/v) (Spherotech) were incubated for 30 minutes with 0.05% BSA to block potential nonspecific EV binding sites prior to use. EV samples labeled with CD9-biotin/Qd655-SAV as described above were mixed with 200 μL of biotinylated beads and diluted to a final volume of 1 mL with PBS. Samples were incubated at room temperature on a tube rotator to ensure continuous mixing. Samples were centrifuged at 14,000 *g* for 5 minutes and the supernatant was carefully aspirated and 450 nm filtered prior to analysis using NTA.

### High-performance liquid chromatography depletion of unconjugated Qd

EV samples were prepared using ultracentrifugation as described above. All experiments were performed using high-performance liquid chromatography (HPLC) instrument Agilent 1100 with an AdvanceBio SEC-5 column (300 Å 2.7 μm, 7.8 × 300 mm, Agilent) with mobile phase PBS (pH 7.4, 0.5 mL/minute), with UV absorbance (DAD) at 220 nm and 280 nm and fluorescence detection (FLD) at 655 nm, and acquired using Agilent software (ChemStation Rev. A.10.02). Representative aliquots containing 1 × 10^9^–1 × 10^10^ EV and/or 0.5-2.0 μL Qd655-SAV were used to measure the retention time of the unlabeled EV population, unconjugated Qd655-SAV, and CD9biotin-Qd655-SAV labeled EV. Based on these retention times, EV fraction collection was scheduled to maximize capture of labeled EV with minimal contamination of free Qd. A second fraction was timed to capture the majority of free Qd. Primary labeling of EV with CD9-biotin was performed as described above. Samples were prepared for HPLC separation using 5 × 10^9^ primary labeled EV (counted on NTA after the primary-Ab wash) and 2 μL Qd655-SAV. Here, excess secondary Ab (compared to standard labeling protocol) was used to allow for greater visualization and recovery of Qd in the HPLC chromatogram and fraction analysis. NTA was performed immediately on the collected fractions.

### Evaluation of immunolabeling efficiency

**Figure 1:**
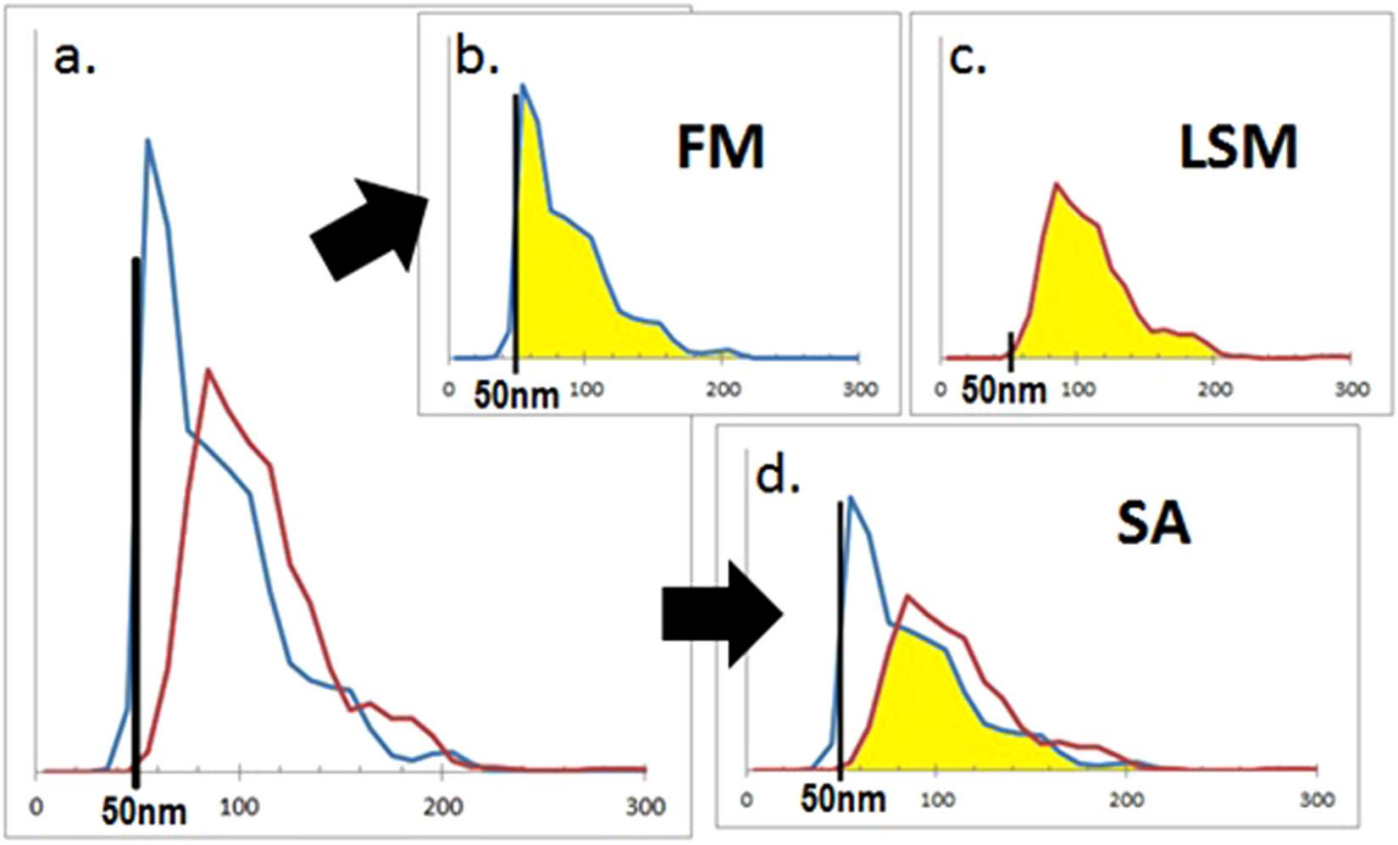
Evaluation of immunolabeling efficiency. Each immunolabeled EV sample was evaluated using NTA in fluorescent (FM, blue line) and light scatter (LSM, red line) modes. (**a**) Paired size distribution histograms plotted on the same axes. (**b**,**c**) The FM:LSM percentage compares the EV populations represented by the shaded areas >50 nm in size in each mode. (**d**) The SA percentage compares the shared (shaded) portion to the total number of particles >50 nm counted in LSM.

Each immunolabeled sample was analyzed using NTA in FM, immediately followed by analysis of the same sample in LSM. The resulting averaged size density histograms were plotted on the same axes to determine the overlap between the two modes (Fig. 1). One evaluation method determined the total count of particles larger than 50 nm for each mode and directly compared the particle number in FM to that in LSM, generating a FM:LSM percentage. A second method evaluated the area shared between the two curves, including only the area where the two detected populations overlapped. This overlapping area was compared to the total number of particles larger than 50 nm counted in LSM to create a shared area (SA) percentage. The 50 nm cutoff was selected to coincide with the lower limit of detection of particles in LSM. For Qd depletion experiments, the comparison was refined to compare FM:LSM or SA in particles ranging in size from 50–200 nm only, to restrict the scope to particles specifically within the EV size range.

### Data reporting and statistics

All data are reported descriptively. For NTA, size distribution histograms are reported as the average of 5 sequential replicate recordings of each individual sample, with error bars representing the standard error of the mean. Throughout this manuscript, data are reported as EV concentration (particles/mL) with standard error to replicate the standard data output from the NTA software. Corrections have been applied for all NTA dilutions, and within Qd depletion experiments, sample aliquots were prepared to ensure equal dilution effects for each arm of the experiment. Normality of data was assessed using Kolmogorov-Smirnov testing. Normally distributed data were compared using the paired Student’s t-test and non-normally distributed data were compared using the Wilcoxon signed-rank test, with significance set at p ≤ 0.05. We have submitted all relevant data of our experiments to the EV-TRACK knowledgebase (EV-TRACK ID: EV180002)^38^.

## Results

### Characterization of EV

**Figure 2:**
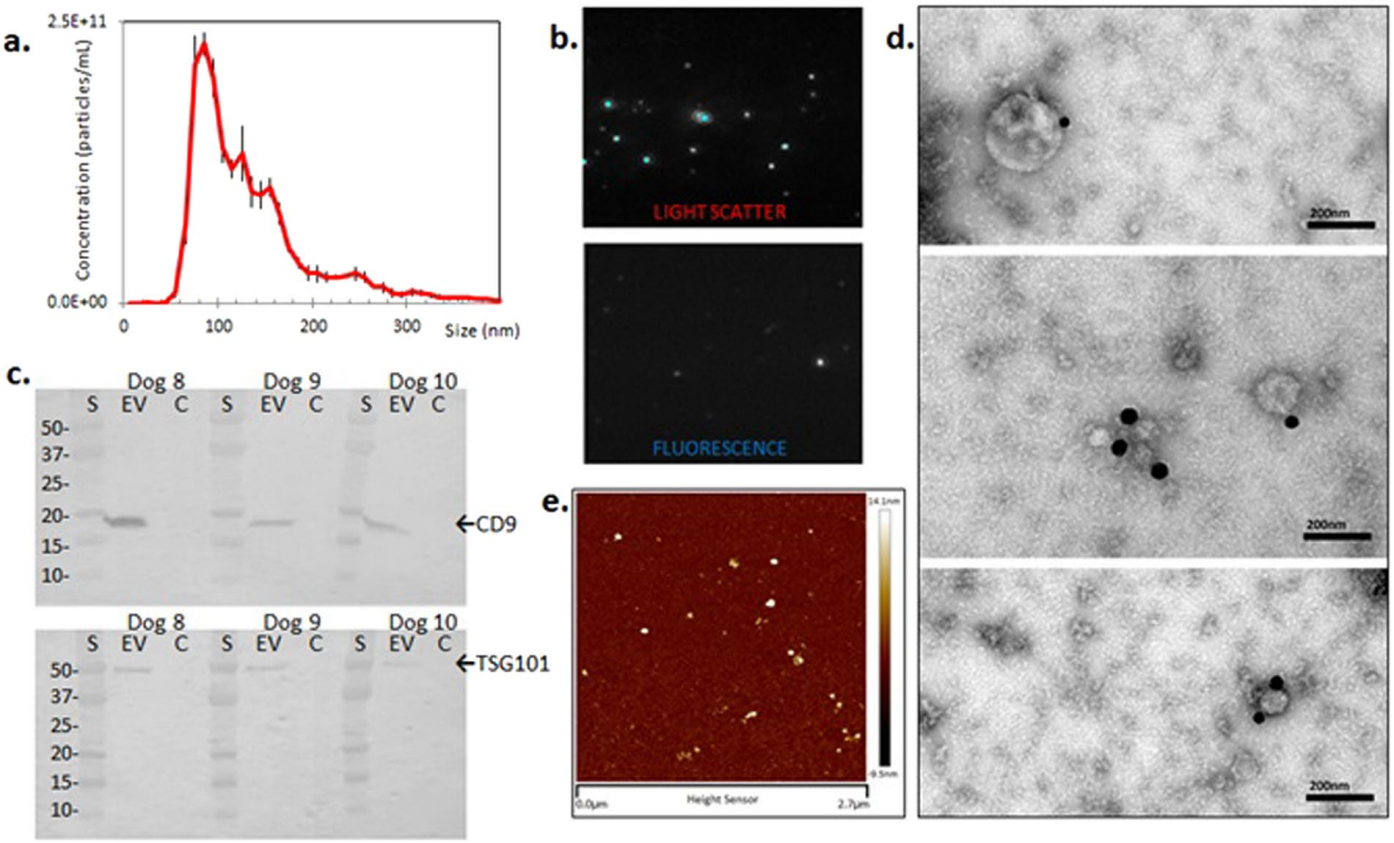
Characterization of EV. (**a**) Average size density histogram of five canine MSC-derived EV samples evaluated in NTA light scatter mode; error bars represent ±1 standard error. (**b**) Appearance of CD9-biotin/Qd655-SAV labeled EV in light scatter (top) and fluorescence (bottom) modes on NTA. (**c**) Western blots of canine EV and parent cells from three different cell lines demonstrating enrichment for CD9 (top, 19 kDa) and TSG101 (bottom, 49 kDa) in only the EV samples. (**d**) Transmission electron micrograph of canine EV labeled with CD9-biotin/streptavidin-gold; gold particles appear black, all scale bars are set to 200 nm. (**e**) Atomic force microscopy of canine EV labeled with CD9-biotin/Qd655-SAV showing a 2.7 μm-wide field.

NTA was used to generate size density histograms of canine placental mesenchymal stem cell EV collected by ultracentrifugation. 86.0% of detectable particles in LSM fell within the size range of 50–200 nm (Fig. 2a). Immunolabeled EV were visible in both LSM and FM (Fig. 2b, Supplemental video 1). Western blotting demonstrated enrichment of characteristic EV proteins CD9 and TSG101 in EV-derived protein samples, relative to each EV sample’s parent cell source (Fig. 2c). Additional western blot analysis was performed on a single EV sample (Dog 11) separated on a density gradient, demonstrating enrichment of EV related TSG101 and CD9 proteins at a density of 1.097–1.127 g/mL (Supplementary Information, part 5). ExoGlow labeling of a representative EV sample (Dog 8) was also consistent with a population highly enriched for small EV based on manufacturer’s claims (Supplementary Information, part 3). Transmission electron microscopy (TEM) confirmed the presence of structures typical of EV in the size range of 40–250 nm and EV labeled with CD9-biotin/streptavidin-gold (Fig. 2d). Atomic force microscopy of EV labeled with CD9-biotin/Qd655-SAV demonstrated a population of discrete singlets, with no appearance of significant EV aggregation (Fig. 2e) that would confound measurements.

### Bead-assisted flow cytometry of EV samples

Bulk labeling of EV demonstrated no evidence of non-specific binding of primary or secondary Ab (Supplementary Information part 1). EV immunolabeling demonstrated CD9 and CD81 expression on surface of EV (Supplementary Information part 1). Direct conjugation of Qd to CD9, direct biotinylation of CD81, and indirect immunolabeling using ms-CD81 with anti-ms-Qd secondary all demonstrated decreased efficacy in immunolabeling experiments (Supplementary Information parts 1,6).

### Single EV immunolabeling evaluated by NTA

Mean and mode particle sizes calculated in FM and LSM for CD9-labeled and isotype-labeled EV samples are summarized in Table 1. In CD9-labeled samples, mean particle size ranged from 128.3–167.5 nm in FM and 149.1–187.0 nm in LSM, demonstrating a trend towards increased mean size detected in LSM compared to FM (p = 0.08). In contrast, the mean particle size for isotype-labeled samples in LSM ranged from 106.0–160.5 nm, consistent with a significantly decreased mean particle size compared to CD9-labeled samples (p = 0.028). The mode particle size for CD9-labeled samples was 90.7–112.0 nm in FM and 101.7–126.5 nm in LSM. These size ranges were significantly different (p = 0.032), with smaller reported mode particle size in FM in all but one sample source (Dog 5, whose mode size values differed by <1%). In isotype-labeled samples analyzed in FM, mean particle size was 20.1–37.9 nm, with mode size ranging from 20.6–26.0 nm, consistent with reported Qd sizes.

**Table 1:** Particle sizes calculated by NTA in fluorescent (FM) and light scatter (LSM) modes.

		Dog 1	Dog 2	Dog 3	Dog 4	Dog 5	HEK-293
**NTA calculation of mean particle size**							
CD9	FM	128.3 ± 3.2	130.5 ± 3.8	134.4 ± 15.6	165.9 ± 8.3	144.3 ± 7.3	167.5 ± 2.4
	LSM	154.7 ± 5.2	156.3 ± 1.9	151.6 ± 7.9	187.0 ± 6.3	149.1 ± 4.7	157.5 ± 4.3
Isotype	FM	31.7 ± 1.8	37.2 ± 3.6	29.6 ± 7.2	37.9 ± 2.9	27.1 ± 1.1	20.1 ± 2.0
	LSM	134.2 ± 3.8	126.7 ± 3.7	136.0 ± 4.2	160.5 ± 6.1	130.3 ± 2.5	106.0 ± 5.2
**NTA calculation of mode particle size**							
CD9	FM	91.0 ± 8.0	90.7 ± 4.0	100.8 ± 4.5	104.1 ± 12.8	102.7 ± 9.6	112.0 ± 15.7
	LSM	104.0 ± 6.6	126.5 ± 6.4	118.8 ± 10.8	108.1 ± 8.1	101.7 ± 7.0	118.6 ± 3.9
Isotype	FM	25.5 ± 0.3	26.0 ± 1.5	26.0 ± 4.1	25.2 ± 0.3	24.4 ± 0.2	20.6 ± 1.3
	LSM	92.4 ± 2.2	96.4 ± 3.2	114.5 ± 5.9	123.6 ± 12.2	121.4 ± 12.6	64.6 ± 0.8

Size characteristics of immunolabeled EV are reported, including mean and mode particle sizes ± standard error.

Analysis of CD9-labeled samples in FM indicated few detectable particles <50 nm (0.03–13.7% of the total count), indicating that most detectable fluorescent particles were associated with EV, evidence that optimal immunolabeling was achieved (Table 2). In comparison, the majority (79.7–94.6%) of particles detected in FM analysis of isotype-labeled samples were <50 nm (Table 2), indicating a significant difference between the particle sizes detected in CD9-labeled and isotype-labeled samples (p < 0.0001). This is consistent with the majority of detectable fluorescent particles being free Qd (20–30 nm) in isotype-labeled samples. For all samples evaluated in LSM, detection of very small particles was negligible, with 98–100% of the detectable particles in LSM being >50 nm (Table 2) demonstrating that neither Qd nor very small biological particles produce sufficient light scatter for detection using this mode.

**Table 2:** Size grouping of particles in fluorescent (FM) and light scatter (LSM) modes.

	Dog 1	Dog 2	Dog 3	Dog 4	Dog 5	HEK-293
**Percentage of total particles detected in FM <50** **nm in size**						
CD9	8.0%	6.1%	13.7%	3.2%	4.4%	0.03%
Isotype	86.2%	79.7%	85.4%	80.4%	88.9%	94.64%
**Percentage of total particles detected in LSM >50** **nm in size**						
CD9	100%	100%	99.5%	100%	100%	100%
Isotype	100%	99.9%	100%	99.9%	100%	98.0%

Percentage of total counted particles in immunolabeled EV samples based on size and immunolabel. For each EV source, all counts of particles <50 nm in size are from FM analysis, while all counts of particles >50 nm in size are from LSM analysis of the same sample.

**Figure 3:**
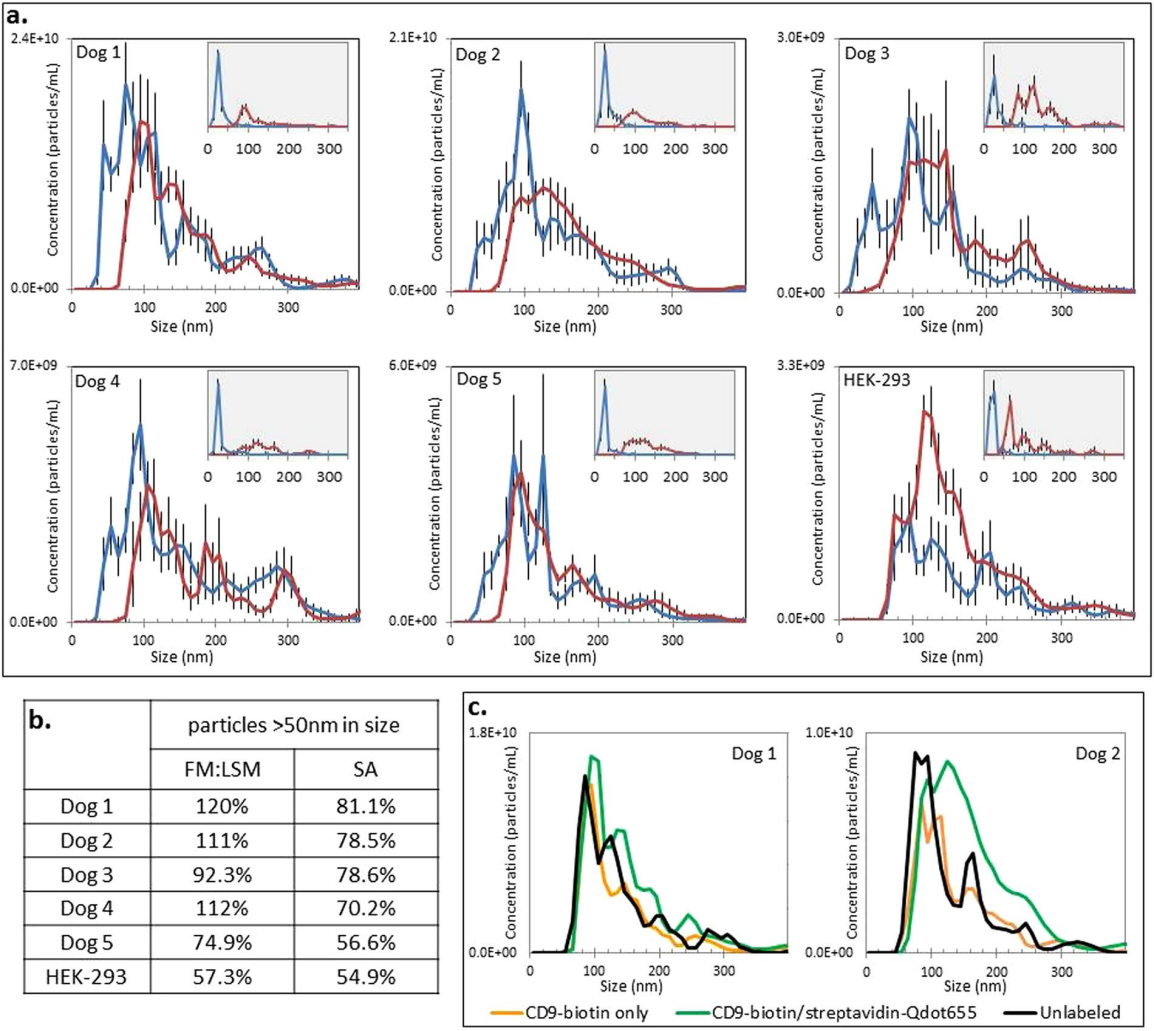
Single EV CD9 immunolabeling using NTA. (**a**,**c**) Particle size distribution of canine and HEK293 cell-derived EV samples assessed using light scatter mode (LSM, red line) and fluorescent mode (FM, blue line); error bars represent ±1 standard error. (**a**) Immunolabeling using CD9-biotin/Qd655-SAV of EV from five different canine cell lines (inset graph depicts each sample’s isotype-biotin/streptavidin-Qdot655 control). (**b**) Percentage of particles >50 nm in size visible in FM compared to the number visible in LSM. (**c**) Light scatter size distribution of unlabeled EV, CD9-biotin labeled EV, and CD9-biotin/Qd655-SAV labeled EV.

Particle size distributions varied between EV sources (different cell lines) (Fig. 3a). Indirect immunolabeling using CD9-biotin/Qd655-SAV demonstrated comparable concordance of particles larger than 50 nm visible in FM and LSM across five different canine EV cell lines, with FM:LSM ranging from 74.9% — >100% and SA from 56.6% — 81.1% (Fig. 3b). EV derived from HEK-293 cells exhibited slightly lower concordance between modes, with FM:LSM 57.3% and SA 54.9% (Fig. 3a,b). Isotype-biotin/Qd655-SAV immunolabeling showed the expected difference between particles visible in FM (majority <50 nm, corresponding with free Qd) and LSM (majority >50 nm, corresponding with EV) (Fig. 3a insets).

Comparison of LSM size distribution histograms of unlabeled, CD9-biotin labeled, and CD9-biotin/Qd655-SAV labeled EV demonstrate a minor increase in calculated EV size associated with the addition of secondary Qd655-SAV (Fig. 3c).

### EV immunolabeling in the presence of non-EV particles

**Figure 4:**
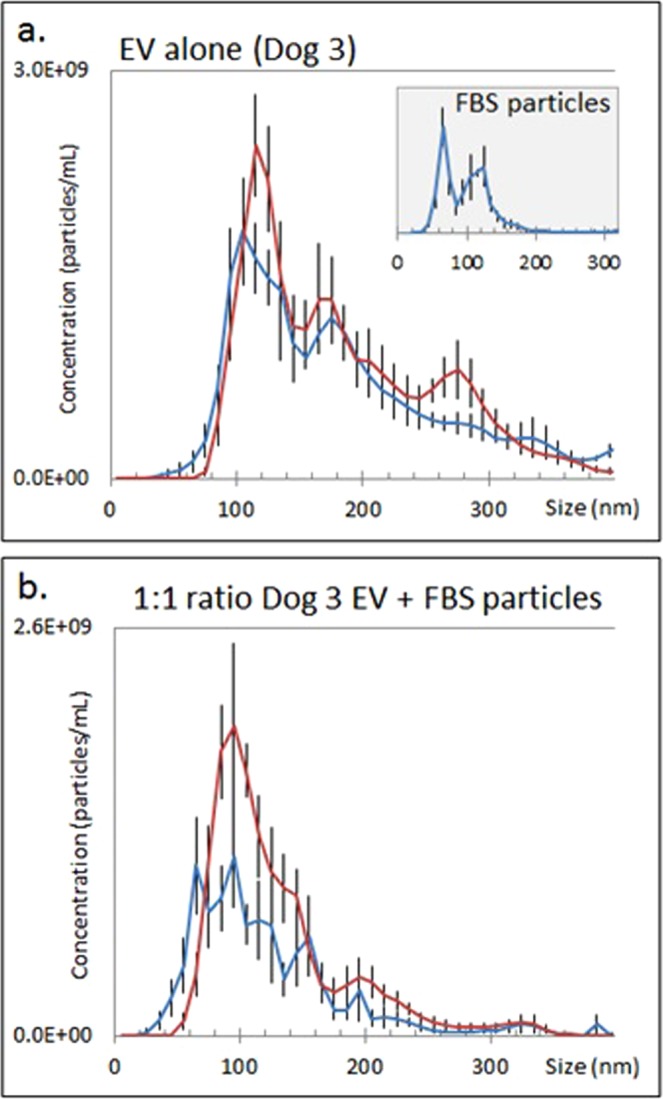
Single vesicle labeling in the presence of non-EV particles. Particle size distribution assessed using light scatter mode (red line) and fluorescent mode (blue line); error bars represent ±1 standard error. (**a**) Size distribution of CD9-biotin/Qd655-SAV immunolabeled EV alone; LSM particle size distribution of FBS-derived particles inset (blue line in this inset graph only). (**b**) Size distribution of 1:1 mixture of isolated EV with FBS-derived particles immunolabeled with CD9-biotin/Qd655-SAV.

Dilution of canine EV was achieved using EV–depleted FBS as a source of non-canine, non-EV proteins. Immunolabeling a 1:1 mixture of canine EV and FBS-derived particles using CD9-biotin/Qd655-SAV demonstrated a decrease in the percentage of particles visible in FM versus LSM when compared to that observed for canine EV only, demonstrating specificity of the EV immunolabeling method (Fig. 4). The FM:LSM decreased from 91.9% (EV alone) to 66.7% (1:1 EV:protein mixture); similarly, the SA decreased from 82.3% (EV alone) to 58.1% (1:1 EV:protein mixture).

### Biotinylated bead-assisted depletion of excess streptavidin-Qd655 during indirect immunolabeling

**Figure 5:**
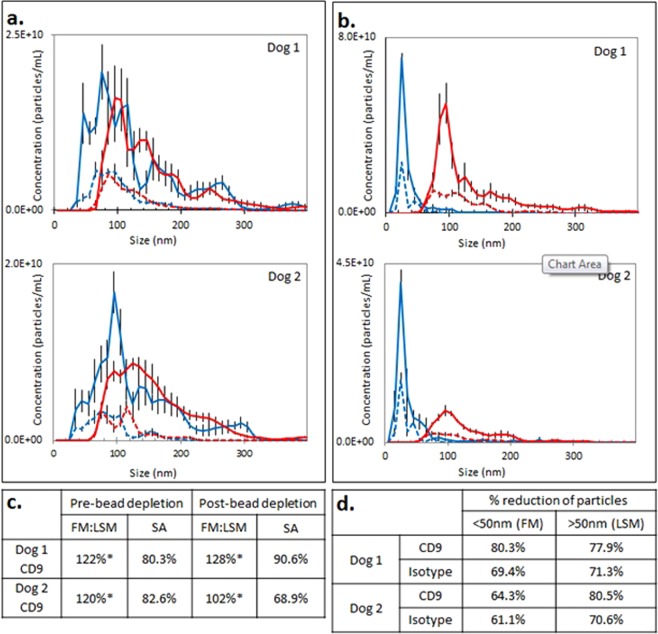
Biotinylated bead–assisted depletion of free Qd655. (**a**,**b**) Particle size distribution assessed using light scatter mode (LSM, red line) and fluorescent mode (FM, blue line) in EV from two canine cell lines. Solid lines indicate particle distribution prior to Qd depletion using biotinylated beads; dashed lines represent particle distribution following depletion. Error bars represent ±1 standard error. (**a**) Immunolabeling using CD9-biotin/Qd655-SAV with subsequent Qd depletion. (**b**) EVs labeled with isotype-biotin/Qd655-SAV with subsequent Qd depletion. (**c**) Assessment of FM:LSM and SA percentages for particles ranging in size from 50–200 nm pre- and post-Qd655-SAV depletion. (**d**) Percent reduction in particle counts following Qd depletion using biotinylated beads. Pre- and post-depletion particle counts were obtained from LSM data for particles >50 nm and from FM data for particles <50 nm.

Use of biotinylated beads was effective in reducing the number of excess unbound Qd associated with immunolabeling using CD9-biotin/Qd655-SAV. Particles counts in the size range of 50–200 nm demonstrated good concordance between FM and LSM before and after incubation with biotinylated beads (n = 2, Fig. 5a), with FM:LSM > 100% in all instances, and SA 80.3–82.6% before and 68.9–90.6% after bead-assisted Qd depletion (Fig. 5c). However, concurrent with depletion of free Qd655-SAV, particles larger than 50 nm were also markedly decreased following Qd depletion (Fig. 5a,b,d). Incubation of biotinylated beads with isotype-biotin/Qd655-SAV demonstrated similar reduction in counts of particles both larger and smaller than 50 nm following bead depletion (Fig. 5b,d). Overall, for both CD9- and isotype-labeled samples, the reduction in total particles smaller than 50 nm ranged from 61.1–80.3%, while the reduction in total particles larger than 50 nm ranged from 70.6–80.5% (Fig. 5d) consistent with pronounced adsorption of EV to beads as a consequence of this process.

### Size-exclusion high performance liquid chromatography (SEC-HPLC) depletion of unbound streptavidin-Qd655 during indirect immunolabeling

**Figure 6:**
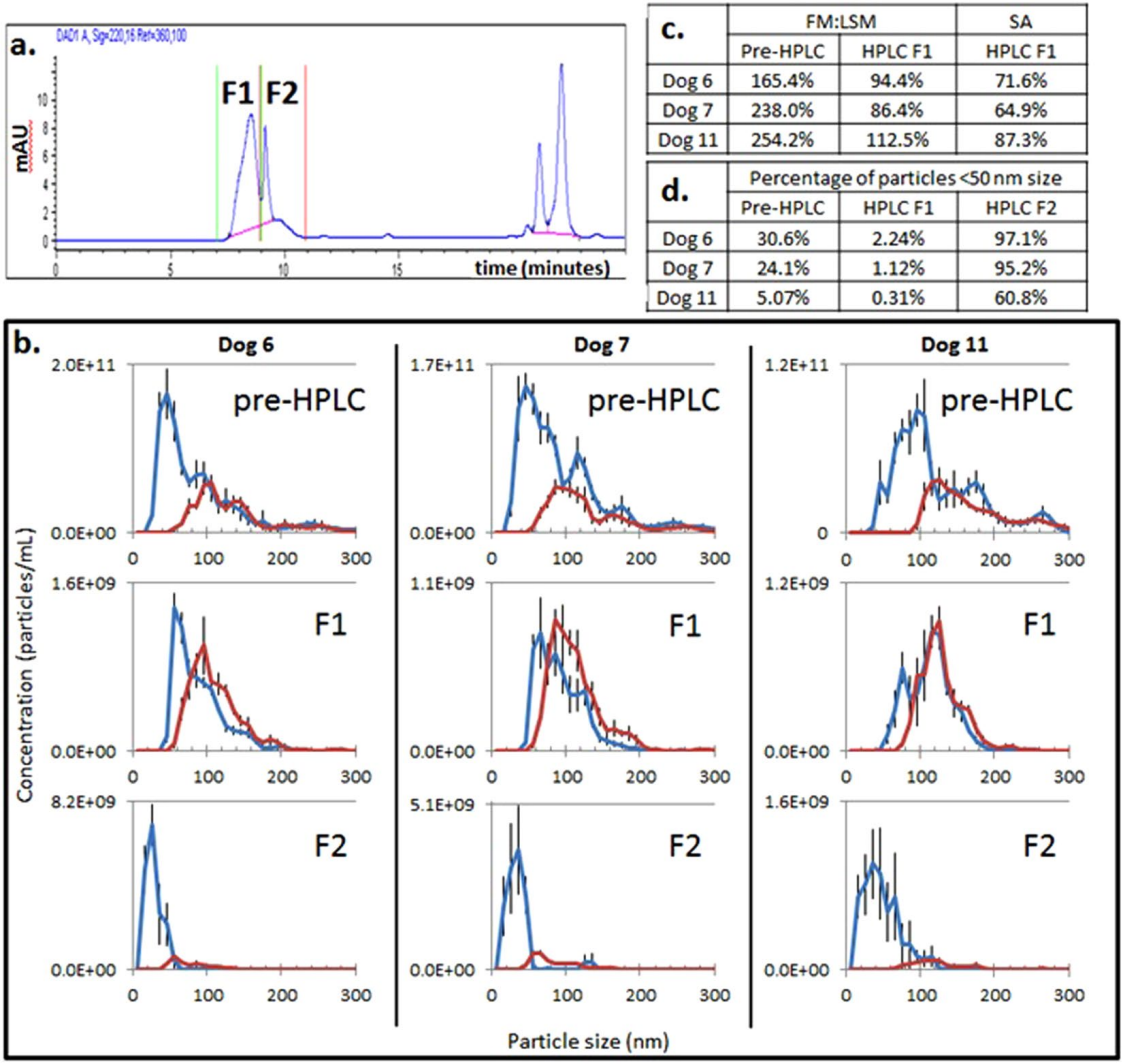
HPLC depletion of free Qd655. (**a**) Chromatogram generated by the HPLC DAD at 220 nm and FLD at 655 nm highlighting the early immunolabeled vesicle fraction (F1), later free Qd655-SAV fraction (F2), and a late peak representing small molecular weight proteins. (**b**) Particle size distribution assessed using light scatter mode (LSM, red line) and fluorescent mode (FM, blue line) in samples prior to separation (pre-HPLC) and within each collected fraction (F1, F2); error bars represent ±1 standard error. (**c**) Assessment of FM:LSM and SA percentages for particles ranging in size from 50–200 nm pre- and post-HPLC separation. (**d**) Percentage of the total sample particle count determined in FM comprised of particles <50 nm in size pre- and post-HPLC separation.

Multiple types of SEC-HPLC columns were found to adsorb Qd (data not shown) consistent with the literature^39^. However, one silica-based column (AdvanceBio SEC, Agilent) with a hydrophilic polymeric coating did not adsorb Qd, enabling separation of Qd on the basis of their unique size and retention time. SEC-HPLC separation of immunolabeled EV consistently demonstrated a fluorescently labeled EV peak in close proximity to (but distinct from) the free Qd655-SAV peak (Fig. 6a). Immunolabeled EV were prepared with excess Qd655-SAV; when viewed in FM, a variable percentage of small particles (<50 nm) was present, ranging from 5.1–30.6% of the total count (Fig. 6b,d). NTA of the immunolabeled EV fraction (F1) demonstrated both a high FM:LSM (86.4– >100%) and SA (64.9–87.3%) for particles ranging in size from 50–200 nm, as well as a markedly reduced proportion of particles <50 nm (0.31–2.24% of the total particle count in FM) (Fig. 6b,c,d). NTA evaluation of the delayed fraction corresponding to Qd (F2) revealed a large proportion of small particles (60.8–97.1% of the total particle count) visible in FM, with few particles visible in LSM (Fig. 6b,d). Using SEC-HPLC, total particles collected in the EV fraction compared to the expected starting amount (5 × 10^9^ immunolabeled EV) varied from 73.4–86.4% (3.7–4.3 × 10^9^ total collected particles in F1), demonstrating good sample recovery.

## Discussion

The results of this study demonstrate that indirect single-EV immunolabeling using Qd within the NTA platform enables rigorous immunophenotypic characterization concurrent with estimation of the bulk concentration and size distributions of particles within a sample. A critique of the immunolabeling method, Qd depletion, and NTA protocols is provided below.

### Assessment of single-EV immunolabeling

Repetitions of the CD9-biotin/Qd655-SAV protocol in both canine- and human-derived EV consistently demonstrated reproducible immunolabeling. Good concordance between NTA-calculated particle size in FM and LSM was observed consistently, with distinct overlap between FM and LSM size distribution histograms. Fluorescent EV-sized particles (>50 nm) were rarely observed in isotype-labeled samples, indicating negligible non-specific EV-Qd binding or Qd aggregation.

The immunolabeling of EV with Qd increased size ranges modestly. This can be observed by comparing the LSM size-distribution histograms, and mean and mode particle sizes between the CD9-immuno-labeled samples and respective isotype controls. We speculate that this is due to binding of Qd655-SAV to labeled EV, the extent of which likely varies with CD9 expression, efficiency of primary and secondary labeling. Further evaluation using TEM or cryo-electron microscopy to quantify the number of bound Qd per EV is warranted to clarify this question.

Immunolabeling of an EV sample that was specifically “contaminated” with FBS-derived particles generated an expected decrease in the number of Qd-labeled EV, based on lower FM:LSM and SA than the “non-contaminated” sample; however, a mixture of equal parts EV and contaminating particles did not exhibit exactly 50% reduction in immunolabeling efficiency. These FBS-derived (EV-depleted) particles were produced by prolonged ultracentrifugation, a process that is not 100% efficient in removing EV. A multi-species reactive antibody against CD9 was used, so it is possible that bovine FBS-derived EV were immunolabeled, obfuscating the observed stoichiometry of this dilution experiment. However, this dilution study effectively demonstrated that Qd immunolabeling is largely dose-dependent. In addition, HEK-293 was the only human-derived cell line selected for EV production; however, HEK-293 are known to elaborate adenovirus (capsid size of 70–100 nm). Given the likelihood that the HEK-293 sample contained EV-sized virus, specificity of immunolabeling EV (and not co-isolated virus) may have contributed to the decreased FM:LSM and SA observed from this source.

The full range of sizes of immunolabeled canine-derived EV prior to Qd depletion consistently included small particles, typically ranging in size from 30–100 nm, that was detectable in FM but pronouncedly less numerous in LSM. These smaller fluorescent particles could be a mixture of free Qd, small Qd aggregates, proteins undergoing nonspecific Ab or Qd interactions, or fluorescently immunolabeled small EV that, due to their size, cannot be visualized in LSM. Technical limitations of the NTA system preclude the ability to detect very small EV or other biological particles with low refractive indices using LSM, while sample heterogeneity may confound detection of smaller particles in a polydisperse sample. In addition to the Qd labels, these factors cause discordance in size ranges between LSM and FM. Advanced imaging (electron microscopy, super-resolution microscopy) could be pursued in additional experiments to investigate heterogeneity of small fluorescent particles that are detected upon fluorescent labeling. However, the presence of small EV in FM did not preclude an accurate comparison of FM and LSM.

Evaluation of immunolabeling efficiency was approached in two ways. The FM:LSM percentage does not presume that either LSM or FM provides a more accurate particle count, and simply compares the generated counts of particles larger than 50 nm calculated in each NTA mode. Logically, only a subset of the immunolabeled EV are expected to demonstrate a targeted phenotype due to inherent heterogeneity of EV. Therefore, the FM:LSM percentage should not exceed 100%. However, the FM:LSM was reported to exceed 100% in some samples in this study, which limits the utility of using this method as an exact quantification of overall phenotype prevalence. Alternatively, the SA percentage used in this study assumes the particle count determined in LSM is the population over which accurate comparisons can be made between LSM and FM. The SA percentage compares the area shared by the histograms of LSM and FM for particles larger than 50 nm. While this consistently yields a SA below 100%, it also excludes a subset of small particles (arguably, immunolabeled small EV) visible only in FM. The cutoff value of 50 nm for both of these methods was arbitrary, based on expected EV size (50–200 nm), the known size of Qd (15–20 nm) which is considerably smaller, and the lower end of particle size detection capability in LSM when the mode is optimized for visualization of biological particles (approximately 50 nm). However, it is important to acknowledge that any size cutoff value or range could be elected to optimize comparisons of the size histograms generated in the two NTA modes. Thus, our methods are not meant to be prescriptive, but rather a foundation for optimizing NTA-fluorescence systems for each EV type encountered by investigators.

The use of a fluorescent dye to mark all EV in a sample (membrane or cytosolic dyes) would be useful to improve the accuracy by which NTA-FL could estimate epitope prevalence of particles smaller than 50 nm utilizing NTA solely in FM; however, this labeling may not provide EV specificity^26,27^ and many fluorophores are insufficiently bright to be detected by NTA at this time.

### Antibody selection and labeling challenges

Indirect, as opposed to direct, immunolabeling was utilized in our protocol in order to exploit the greater fluorescent signal amplification with indirect labeling. Attempts to biotinylate antibodies, conjugate antibodies with Qd, and apply secondary species-directed anti-IgG antibodies were ineffective strategies in NTA fluorescence measurements in this study. In contrast, commercially prepared biotinylated CD9 antibodies performed well in all experiments which led to the conclusion that biotin-streptavidin Qd is a superior approach to NTA-FL.

One limitation of this method in its present form is the inability to characterize multiple epitopes in a single EV simultaneously. Theoretically, NTA systems permit detection of multiple fluorochrome emissions through use of targeted band-pass fluorescence filters, enabling detection of subphenotypes of EV, but optimal immunolabeling methods must be developed beyond the singular use of indirect labeling with SAV-Qd to take advantage of this strategy.

For biological samples, optimizing primary antibody and Qd requirements is challenging. For the purpose of phenotyping, the ideal quantity of primary antibody is initially unknown. Western blotting or mass spectroscopy could be performed to determine the relative amount of a target protein within an EV population. Using an excess of primary Ab saturates the protein targets, but may increase non-specific binding. The more significant challenge is to reduce excess unbound Qd. As EV and Qd both sediment with comparable ultracentrifugation conditions, differential centrifugation is ineffective for separation of free Qd. Also, the similar size of immunolabeled EV and unbound Qd precludes the use of ultrafiltration to separate the two populations. Hence, size-exclusion chromatography (HPLC) and biotinylated bead-assisted depletion were evaluated as methods to separate free Qd from Qd-labeled EV.

### Biotinylated bead-assisted depletion of excess Qd

Incubation with biotinylated beads was partly effective in reducing the number of unconjugated Qd but markedly reduced recovery of EV, which could be a substantial limitation to downstream applications. We speculate that since streptavidin is able to bind to multiple biotin molecules, biotinylated beads might also bind the SAV-Qd within a Qd-labeled EV complex and remove these from solution. We also observed that non-fluorescent particles larger than 50 nm were reduced following bead incubation in isotype-labeled samples, implicating non-specific binding of EV to the biotinylated beads as another possible cause of larger particle loss. Additionally, beads must be entirely removed from the Qd-depleted EV sample to be analyzed for NTA. Polystyrene beads are relatively large with a high refractive index, thus even rare beads remaining in the Qd-depleted sample may confound accurate particle counts. Further, samples required centrifugation and filtration to clear residual beads prior to evaluation using NTA, likely contributing to loss of EV in Qd-depleted samples. The number of beads used in these experiments was calculated to exceed the binding requirements of the excess Qd present in isotype-labeled samples (Supplementary Information part 7); despite this, only a fraction of the unbound Qd was removed during this depletion step. Therefore, reducing the number of beads to minimize unintentional EV depletion is not practical.

Despite the reduction in both large and small particles following bead-assisted Qd depletion, good concordance between counts obtained in LSM and FM were observed, indicating that use of biotinylated beads may be an effective method for depletion of excess Qd if labeled EV loss is tolerable within an experiment.

### HPLC-facilitated depletion of excess Qd

Size-exclusion chromatography is an isolation method that has been reported to yield an enriched fraction of EV^40,41^. Furthermore, past studies showed that size exclusion chromatography is useful to deplete unconjugated fluorochromes such as CFSE^27^. In this study, HPLC separation offered a robust method to deplete excess Qd from immunolabeled EV samples and thus improve the accuracy to compare LSM and FM. The collected EV fraction repeatedly demonstrated excellent (>90%) concordance between FM and LSM counts for particles ranging in size from 50–200 nm, with negligible contamination of Qd-sized fluorescent particles showing rigorous separation. Collection of a second (delayed) fraction may be pursued to confirm presence of free Qd, but is not a necessary component of the depletion protocol.

Multiple types of silica-based HPLC columns were evaluated prior to identifying the suitability of the Agilent AdvanceBio SEC column, which has a unique column chemistry that minimized sample loss caused by EV and Qd binding to the column. This method appears to be able to separate a relatively large number of excess Qd from immunolabeled EV, although the limits of Qd input have not been rigorously evaluated.

Unlike bead-assisted depletion of excess Qd, this protocol is limited to those with access to specialized equipment. Additionally, this method yields a sample that may be significantly diluted compared to its starting characteristics, depending on the HPLC collection settings employed, which might require concentration of the EV fraction for certain downstream applications. This method also fails to discriminate between labeled EV and other particles within the same size range (large aggregates of protein or Qd) within the collection fraction, though advanced microscopy (TEM) could assist in identifying possible contamination within the EV fraction.

### Optimizing NTA fluorescence settings

Biological samples generate unique challenges to the utility of NTA. EV isolated from cell culture conditioned medium and biofluids can be expected to exhibit polydispersity^42^, which necessitates method adjustments based on individual EV sample characteristics. In light scatter mode, EV can routinely be visualized using only the upper end of the camera sensitivity (level 10 and higher). It is critical to identify a level at which the smallest particles in a polydisperse sample are sufficiently illuminated to be visible to the camera, while avoiding oversaturation of the background and interference from large, bright particles, which overshadow the smaller particles and exhibit halo effects^43^. There is also a discrete lower limit to the detectable size of particles based on their refractive indices^44^. Thus individual Qd, while undetectable in LSM, become traceable when only fluorescent light is tracked by the SCMOS camera (Supplementary Video 1). This phenomenon may be unintentionally exploited due to the size difference of the free Qd and the immunolabeled EV. Small, rapidly moving free Qd become difficult to track when overshadowed by larger, fluorescently labeled EV. In this way, the detectable count of free Qd is decreased in a sample with a high percentage of labeled particles, reducing the “background noise” that could be generated by small numbers of free Qd.

Following initial video capture of a sample, the detection threshold (DT) must be set for software analysis of the sample. Determining a single setting for all samples is impossible, and sample-by-sample optimization of this and other settings may improve NTA measurement reliability^36^. Fundamentally, the lower the DT setting, the greater the sensitivity of NTA to identify small, minimally refractive particles, at the expense of generating increased background noise. Applying different DT in sequential analysis of a single experiment demonstrates variability in total particle count and size distribution as the detection sensitivity is altered (Supplementary Information part 8). If exact particle counts are required, the DT may be “tuned” to a target value established through a corroborative sizing and counting method. If relative proportions are more important, then the DT can be selected based on the desired range of sensitivity to smaller/dimmer (lower DT) or larger/brighter (higher DT) particles.

Parameters may be standardized within each mode to minimize variation in sample analysis. However, the detection requirements for each mode differ; within a single sample, optimal LSM settings will not detect low-level fluorescent particles when applied to FM, while settings optimized for FM will yield an unacceptable amount of noise in LSM analysis. In these experiments, the DT was optimized to maximize identification of small, minimally fluorescent particles (including free Qd).

Quantum dots are ideally suited to NTA applications due to their remarkably stable fluorescence emission, while organic fluorophores with similar excitation and emission spectra may be limited by photobleaching. Unlike flow cytometry, which requires a brief period of fluorescence emission and utilizes a photomultiplier tube to enhance minor signals, NTA requires a prolonged period of stable fluorescence emission for particle tracking to occur. The use of Qd overcomes photobleaching, and to our knowledge is the only fluorochrome that is sufficiently bright and photostable to be useful for NTA-FL at this time. Development of additional photostable fluorochromes for EV immunolabeling would be a significant advance in NTA-fluorescence.

### EV Immunofluorescence applications and future directions

Routine characterization of EV surface marker expression may be of increasing importance as a method to assess uniformity between EV samples, as changes in cell culture parameters and EV isolation methods have been demonstrated to alter the characteristics of collected EV^45,46^. This method enables comparison of samples which differ in origin, time of collection, storage conditions, or that have undergone different isolation, concentration, and purification protocols. In addition, EV-specific immunolabeling provides a method to discriminate fluorescently-labeled EV from contaminating non-EV particles, which can be coupled with complementary evaluations of sample purity, including EV/protein ratios, immunoblots, and TEM. Using this method, expression of specific phenotypes can be directly assessed in conjunction with particle size, which may allow for greater characterization of EV subpopulations, and could highlight focused expression of low-frequency epitopes in specific subsets of the EV population.

Further refinement of the fluorescent immunolabeling technique is warranted, focusing on scaling down input requirements for samples with low EV counts, for example. Evaluation of a larger field of possible antibody candidates may be pursued for analysis of human- or mouse-derived EV. As NTA fluorescence detection technology continues to improve to reduce photobleaching, the utility of organic fluorophores and targeted membrane and cytosolic fluorescent dyes may significantly increase the possible applications of this method, yet pose new challenges in removal and discrimination of unconjugated dyes. Exploration of additional data concurrently generated by NTA (such as fluorescence intensity related to particle size) may provide further insights into the relative frequency of surface marker expression within subsets of the EV population. Comparisons between NTA-FL and nano-FACS are warranted in particles with similar size ranges to understand better the utility of these methods.

In conclusion, the described EV immunolabeling method provides a reliable, complementary technique that can be readily incorporated into existing NTA protocols to provide particle concentration, size distribution, and surface phenotype of EV samples. These insights will advance our understanding of EV heterogeneity and further rigor and transparency of EV experiments.

## Supplementary information

Supplement Video 1

Supplementary Data
