# Supplementary material for: Improved methods for fluorescent labeling and detection of single extracellular vesicles using nanoparticle tracking analysis
Source: Sci Rep. 2019 Aug 23;9:12295. doi: 10.1038/s41598-019-48181-6 (PMC6707283; doi:10.1038/s41598-019-48181-6)

**SUPPLEMENT: Improved methods for fluorescent labeling and detection of single extracellular vesicles using nanoparticle tracking analysis**

Kristen E. Thane, Airiel M. Davis, and Andrew M. Hoffman

Department of Clinical Sciences

Cummings School of Veterinary Medicine

Tufts University

Corresponding author:

Andrew Hoffman, Cummings School of Veterinary Medicine, Tufts University, 200 Westboro Rd, Building 21, Room 102, North Grafton, MA 01536

Phone: 508-887-4589

Fax: 508-887-4363

Email: [andrew.hoffman@tufts.edu](mailto:andrew.hoffman@tufts.edu)

Support: Shipley foundation.

**SUPPLEMENT**

**Part 1: Bulk Labeling of EV (bead assisted bulk flow cytometry)**

**Method:** Particle concentrations were established for each unlabeled EV sample. A volume containing 1x10^10^ particles was incubated with 10 μL 3.9 μm latex beads (corresponding to 1.2x10^7^ total beads) at room temperature for 15 minutes prior to diluting the sample to a total volume of 1 mL with PBS and incubating on a tube rotator at room temperature for a total of 120 minutes. EV-coated beads were pelleted by centrifugation at 1,500 *g* for 3 minutes and 1% BSA was added in a total volume of 500 μL for 30 minutes to block any unbound sites on the beads. The beads were pelleted and resuspended in a final volume of 100 μL of 0.1% BSA. 10 μL of this preparation (approximately 1x10^6^ EV-coated beads) was used to test each primary antibody (Ab). Aliquots of EV-coated beads were incubated for 30 minutes with 1 μg or “one test” of each primary Ab (listed in the methods section) and washed once with 0.1% BSA. Secondary labeling with either 1 μL Qd655-streptavidin (Qd655-SAV) or 1 μL Donkey anti-Mouse IgG-Qd655 was performed. All samples were analyzed using an Accuri C6 cytometer (BD Biosciences), with a minimum count of 50,000 events per sample. Data were analyzed using CFlow Plus Analysis software.

**Results:** Labeling of EV-coated beads showed no significant difference between isotype controls and incubation with Qd655 alone (Figure S1a), demonstrating no evidence of non-specific primary or secondary Ab binding interactions. Characteristic surface epitopes (CD9, CD81) were detected using CD9-biotin/Qd655-SAV and CD81-APC, respectively (Figure S1b,d). Indirect labeling utilizing mouse anti-CD81 (primary) and donkey-anti-mouse-Qd655 (secondary) labeling yielded minimal increase in signal (Figure S1c), suggesting no augmentation of detection using this secondary antibody, as all samples demonstrated positive labeling using CD81-APC (Figure S1b). In contrast to indirect labeling using CD9-biotin/Qd655-SAV, immunolabeling using directly conjugated CD9-Qd655 (SiteClick labeling kit) yielded very low levels of labeling (Figure S1d). This implies that the Qd conjugation reaction was either unsuccessful or damaged the function of the CD9 antibody. Indirect labeling using CD81-biotin (EZ-link biotinylation kit)/Qd655-SAV also demonstrated no difference in signal compared to isotype controls (data not shown).

**Figure S1. Bead-assisted flow cytometry of EV.** Individual sample identification legends are provided for each overlay below. All histograms represent EV adsorbed to beads and labeled in bulk. (a) EVs labeled with isotype or incubated directly with Qd655-SAV. (b) EVs labeled with directly conjugated CD81-APC. (c) EVs labeled with MsCD81/donkey-anti-Ms-Qdot655. (d) Comparison of isotype, directly conjugated CD9-Qd655, and indirectly labeled CD9-biotin/Qd655-SAV populations in two canine and one human-cell derived EV samples.


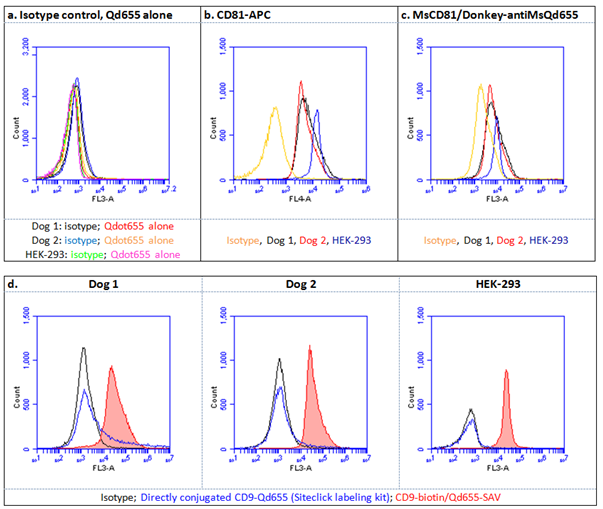


**Part 2. Determination of primary antibody requirements**

**Method**: Bulk immunolabeling was performed as described in Supplementary Information part 1. Samples were prepared by adsorbing varying amounts of EV (from 5x10^9^–1x10^11^) to polystyrene beads and subsequently immunolabeling using 1 μg CD9-biotin with a streptavidin-APC secondary label.

Results: Results are shown in the Figure S2, with 1x10^10^ EV selected as the sample with the best positive signal when using 1 μg CD9-biotin primary immunolabeling.


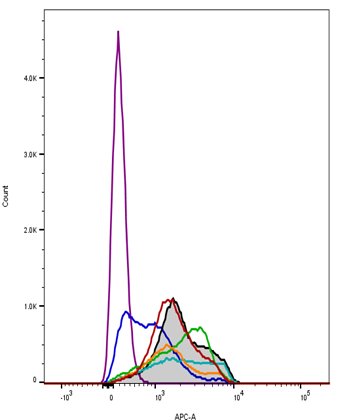


**Figure S2. Determination of primary antibody requirements.** EV immunolabeling assessed using flow cytometry.

Sample legend:

Beads alone (no EV)

5x10^9^ EV

1x10^10^ EV (shaded grey area)

2x10^10^ EV

5x10^10^ EV

7.5x10^10^ EV

1x10^11^ EV

**Part 3: ExoGlow labeling of liposome standards and EV**

**Method:** An ExoGlow labeling kit was obtained from System Biosciences. Liposome standards were labeled per the manufacturer’s directions. Aliquots of ExoGlow-labeled liposomes were diluted in sterile PBS to two discrete concentrations falling within the optimal detection range of 1-10x10^8^ particles/mL (“HIGH” and “LOW”). Each sample was analyzed using NTA as described in the main text, with camera level set at 13 for all LS recordings and at 16 for all fluorescence mode (FM) recordings to maintain uniformity between analyses of the liposomes. Each experiment (set of 5 videos) was analyzed twice, using lower and higher sensitivity settings (detection threshold settings of 4 and 3 for FM recordings, and 5 and 4 for light scatter mode (LSM) recordings) and the resultant size distribution histograms were plotted (Figure S3a). Particle counts were compared within each analysis method (LSM or FM) and between modes to assess similarities between recordings and to evaluate the efficiency of liposome standard labeling as measured by the ratio of particles visible in FM compared to those detected in LSM. These comparisons were made for both the entire size range of particles as well as a subset of particles ranging from 50-200 nm. An aliquot of Dog 8 EV containing 8.3 μg protein (as determined by BCA) was subsequently labeled with ExoGlow per the manufacturer’s directions and analyzed using NTA in FM and LSM.

**Results:** The size distribution histograms of the ExoGlow labeled liposome standards are presented in Figure S3a. The comparison of NTA particle counts obtained using different DT parameters are summarized in Figure S3b. No guidelines are provided by the manufacturer for interpretation of NTA data. For both concentrations of liposomes visualized in LSM, the decrease in detection threshold sensitivity had minimal impact on the total number of particles visualized, with 95-96% of particles detected using the lower sensitivity processing (DT5) compared with higher sensitivity processing (DT4). In contrast, decreasing the sensitivity of the detection threshold in the fluorescence measurements resulted in a decrease in the total number of detectable particles, with only 32-52% of particles detected using the lower sensitivity processing (DT4) compared to higher sensitivity processing (DT3), indicating that a narrow detection window exists for high-sensitivity identification of particles fluorescently labeled with ExoGlow. Concordance between particle counts detected in FM compared to LSM was high whether evaluating all particle sizes (98–105%) or the subset of particles in the 50-200 nm size range (90–96%) when comparing the higher sensitivity detection (DT3) in FM to either detection level in LSM. Unsurprisingly, concordance between modes is poor when comparing the lower sensitivity detection (DT4) in FM to LSM counts. Further evaluation of concordance between modes using liposome standards of varying sizes is warranted to explore differences in labeling affinity associated with EV size and polydispersity.

An aliquot of Dog 8 EV labeled with ExoGlow demonstrated good concordance between size distribution observed in both modes, with FM:LSM 95.2% for all particles counted, 86.8% for particles >50 nm, and SA 67.5% for particles 50-200 nm, suggesting robust enrichment of EV within the detected particle population. As this labeling kit is optimized for the 488 nm laser module for NTA, performing indirect immunolabeling on an ExoGlow labeled sample may not be practical, as it would require selection of a secondary fluorphore with a distinctly different emission spectrum coupled with finely-tuned band-pass filters to allow discrete measurements of each labeling method. However, an aliquot of sample could be labeled with ExoGlow as a method to corroborate EV enrichment prior to pursuing immunolabeling.

Both concentrations of ExoGlow labeled liposome standards and the ExoGlow labeled EV demonstrated a peak of smaller vesicles (approximately 40-70 nm in size) visible in FM that are not detectable (or to a markedly lesser degree) in LSM (Figure S3a,c). This recapitulates a disparity in particle size distributions observed between LSM and FM evaluation of samples labeled with CD9-biotin/Qd655-SAV, which is further discussed in the main text.

**Figure S3. Particle size distribution of ExoGlow labeled samples.** (a) Size distribution of particles in the low concentration (top) and high concentration (bottom) dilutions of liposome standard using low and high detection threshold (DT) analysis settings (legends present on individual figures; error bars represent ±1 standard error). (b) Evaluation of liposome particle counts generated within light scatter (LS) and fluorescence (F) modes comparing within-mode difference between DT levels (F 4/3 and LS 5/4) and comparing each pair of modes using all permutations of more- and less- sensitive DT levels (F3/LS4, F3/LS5, F4/LS4, F4/LS5). (c) Particle size distribution of an ExoGlow labeled EV sample (Dog 8) assessed using light scatter mode (red line) and fluorescent mode (blue line); error bars represent ±1 standard error.

**
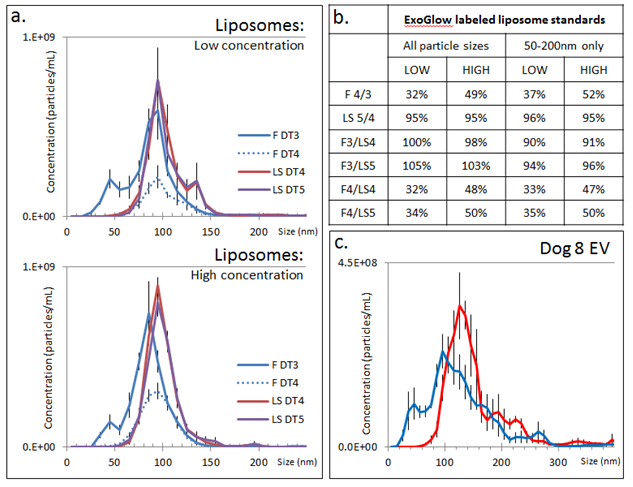
**

**Part 4. Calculation of Qd requirements**

**Method:** Primary immunolabeling of a sample of canine MSC-derived EV was performed as described in the methods section of the main text. Varied amounts of Qd655-SA (from 0.5–5 μL) were incubated with aliquots of this primary labeled EV sample. All samples were evaluated using NTA to determine particle size distribution histograms in light scatter mode (LSM) and fluorescent mode (FM). Particle calculation for the 0.5 μL Qd sample was analyzed with detection thresholds (DT) of 3 and 10; all other samples were analyzed with DT10 due to background interference from free Qd at lower DT settings.

**Results:** Increasing numbers of particles <50 nm (corresponding with unbound Qd) was observed with larger volumes of Qd used, with less than 2.5% of total detected particles <50 nm in samples labeled with 0.5 μL Qd, whereas 21.5–38.8% of total detected particles were <50 nm in samples labeled with 2–5 μL Qd, despite the high DT employed (Figure S2). The FM:LSM was >100% for samples labeled with ≥2 μL Qd.

**Figure S4. Calculation of Qd requirements.** Particle size distribution histograms in light scatter mode (LSM, red lines) and fluorescent mode (FM, blue lines) for a canine MSC-derived EV sample primarily immunolabeled with CD9-biotin and incubated with different volumes of Qd655-SAV. Increasing numbers of particles smaller than 50nm (consistent with unbound Qd) can be observed with Qd volumes ≥2μL. Calculations were performed with detection thresholds (DT) of 3 and 10 for the 0.5μL Qd labeled sample, but could only be performed with DT10 due to increased background noise with lower DT level in all other samples.

**
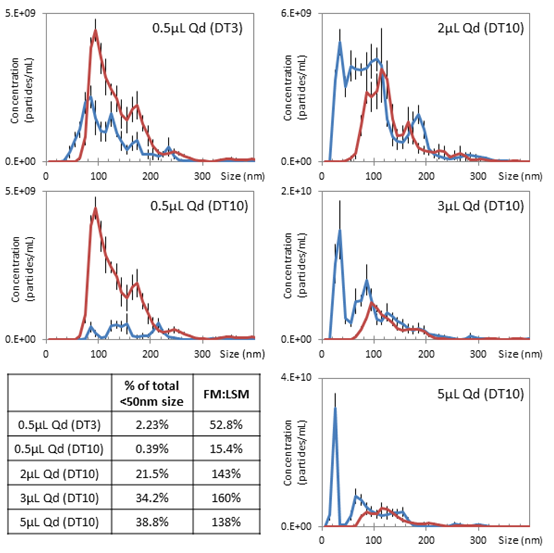
**

**Part 5: Density gradient and Western Blot analysis of EV**

**Method:** An aliquot of 1x10^11^ EV was loaded at the bottom of a discontinuous (10-20-30-40%) iodixanol gradient in a 0.25M sucrose buffer and ultracentrifuged at 350,000 *g* for 2 hours using a SW-55 Ti rotor (*k-*Factor 48, Beckman Coulter). 625 μL fractions were aspirated from the top of the gradient and density was calculated based on absorbance at 340 nm. Fractions were concentrated using a 10 kDa molecular weight cutoff filter (Amicon) and normalized to the largest recovered volume. Particle counts (NTA) and protein quantification (BCA) were performed as described in the methods of the main text. Equal volumes of the concentrated fractions were loaded for Western Blot analysis as described in the main text.

**Results:** The majority of particles were observed in fractions 1 through 4, corresponding with a density range of 1.090–1.127 g/dL (Figure S5a). The greatest intensity of signal on the Western Blot was observed for both TSG101 and CD9 in fractions 2 and 3 (density 1.097–1.118 g/dL), with detectable but less intense signal observed in fractions 1 and 4 as well (density 1.090 g/dL and 1.127 g/dL, respectively).

**Figure S5. Density gradient separation and Western blotting.** (a) Total particle counts and protein amounts obtained from each gradient fraction (fractions concentrated to equal volume using a 10 kDa molecular weight cutoff filter). (b) Regions of interest of Western blots for TSG101 (top) and CD9 (bottom) of the concentrated gradient fractions.

**
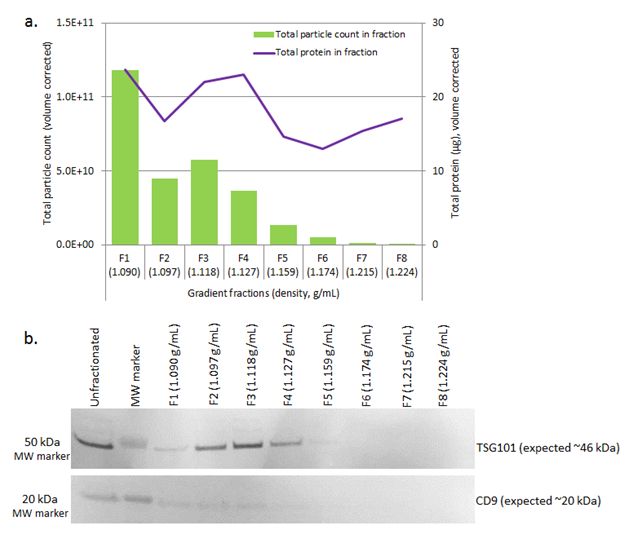
**

**Part 6: Additional EV labeling with NTA evaluation**

**Method:** EV were immunolabeled as described in the methods section using biotinylated CD81 (biotinylated using a Thermo Fisher EZ-Link Micro Sulfo-NHL-biotinylation kit) or directly conjugated CD9-Qd655 (direct conjugation performed using a Thermo Fisher SiteClick Qd655 Antibody Labeling Kit).

**Results:** Indirect immunolabeling of EV using mouse anti-CD81 and donkey-anti-mouse-Qd655 resulted in a small fraction of particles positive for CD81 with both canine EV (FM:LSM 11.9%, SA 11.3%) and HEK-293 EV (FM:LSM and SA 14.5%) (Figure S6a). EV immunolabeling using directly conjugated CD9-Qd655 (SiteClick labeling kit) also demonstrated a low fraction of CD9-positive EV in two canine cell lines (Dog 1: FM:LSM 5.1%, SA 3.5%; Dog 2 FM:LSM 5.98%, SA 4.34%) (Figure S6b). In contrast, CD9-biotin/Qd655-SAV immunolabeling established much higher proportion of CD9-positive EV from the same sources, with FM:LSM >100% and SA 78.5-81.1% (Figure 3b in main text). These findings coincide with the results obtained with bulk immunolabeling experiments (Supplementary information part 1).

**Figure S6. Single EV immunolabeling with alternative Ab using NTA**. Particle size distribution assessed using light scatter mode (red line) and fluorescent mode (blue line); error bars represent ±1 standard error. (a) EV labeled with msCD81 primary and donkey-anti-Ms-Qd655 secondary Ab. (b) EV labeled with directly conjugated CD9-Qd655 (Siteclick kit, Thermo Fisher).


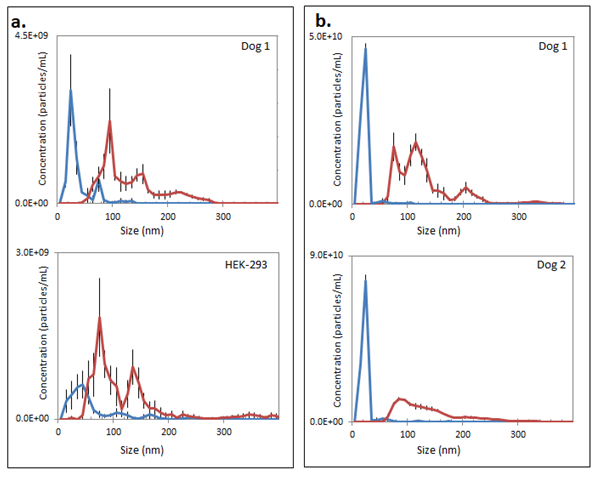


**Part 7. Calculation of biotinylated bead requirement**

Per the included data sheet for 1% 0.74 nm biotinylated beads, 1 mg of beads can bind 30 μg avidin. The number of beads is not provided, nor is the number micrograms of streptavidin associated with the conjugated Qd. To assess binding utility, the binding capacity was converted to potential binding of units of avidin (assuming an equal relationship for capability of binding of units of streptavidin).

Molecular weight of Avidin: 67,000-69,000 g/mol

[0.00003 g binding capacity]/ [69,000 g/mol] = 4.35 x 10^-10^ mol of avidin bound by 1 mg of beads

[4.35 x 10^-10^ mol] x [6.02 x 10^23^] = 2.61 x 10^14^ units of avidin bound by 1 mg of beads

Qdots are supplied as a 1 μM solution. 0.5–1 μL of Qd is used per experiment.

1 μL x 1 μM = [1 x 10^-12^] x [6.02 x 10^23^] = 6.02 x 10^11^ Qd in 1 μL

However, each Qd has 5-10 streptavidin molecules, and each streptavidin is capable of binding up to 4 biotin molecules, thus:

[6.02 x 10^11^ Qd] x [10 streptavidin/Qd] x [4 binding sites/streptavidin] = 2.41 x 10^13^ maximum possible binding events in 1 μL Qd.

Though it is not specified, it is very unlikely that each biotinylated bead contains only one biotin molecule, implying that the total number of beads available to interact with free Qd is <2.61 x 10^14^, and each bead could theoretically bind multiple streptavidin-Qd.

The volume of biotinylated beads used (200 μL) should have the capacity to capture 5.22 x 10^14^ units of avidin, which is approximately 20 times higher than the maximum possible expected binding events provided in the volume of streptavidin-Qd used in these experiments. This volume was chosen to provide an excess of binding capacity to achieve maximum depletion of free Qd. This volume also accommodated concurrent experimentation using equal volumes of alternative biotinylated bead types with much lower reported binding capacities (alternative bead depletion data not shown).

**Part 8. Different detection thresholds (DT) lead to different total particle counts**

**Method:** EV immunolabeling using CD9-biotin/Qd655-SAV was performed as described in the main text. Analysis of the 5 replicate videos captured in fluorescence mode (FM) was performed using detection threshold (DT) settings from 3 through 10 to highlight the differences in particle counts at each DT level.

**Results:** Following video recording of a sample on NTA, a DT must be selected to obtain particle concentration and generate a size distribution histogram. In FM, lowering the DT allows for greater sensitivity in detecting very small (consistent with free Qd) and small-medium (consistent with immunolabeled EV) fluorescent particles (Figure S8a). Here, increasing the DT setting from 3 to 4 results in approximately 70% of the total particles being counted using DT4 compared to DT3, while only 25% of the particles smaller than 50nm could be identified in DT4 compared to DT3 (Figure S8b). Increasing the DT has the most profound effect on decreasing the detection of the smallest particles in the sample, whereas the detection of particles larger than 100nm is least affected.

**Figure S8. Increasing detection threshold (DT) leads to decreasing particle counts.** (a) Particle concentrations from a single CD9-biotin/Qd655-SAV immunolabeled EV sample evaluated in FM on NTA and analyzed with DT ranging from 3 to 10. (b) Percentage of concentration in DT4 to DT10 compared to that obtained using DT3; all data obtained from a single sample analyzed multiple times using the listed range of DT.


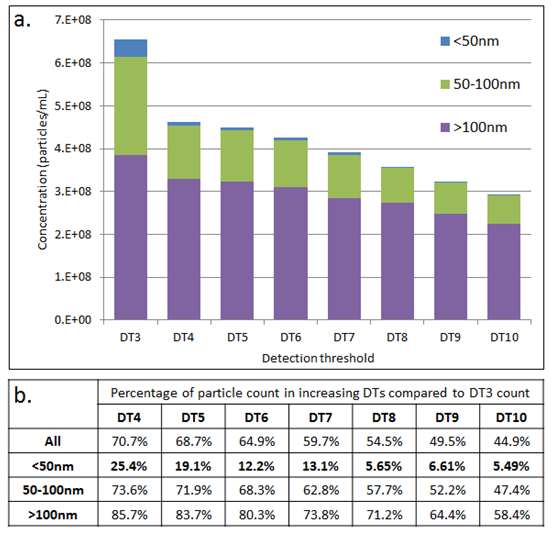

Supplement: Supplementary file 2 — Supplementary Data [file 41598_2019_48181_MOESM2_ESM.docx]
